# Digital Resilience in Dealing with Misinformation on Social Media during COVID-19: A Web Application to Assist Users in Crises

**DOI:** 10.1007/s10796-022-10347-5

**Published:** 2022-10-24

**Authors:** Stefka Schmid, Katrin Hartwig, Robert Cieslinski, Christian Reuter

**Affiliations:** TU Darmstadt, Science and Technology for Peace and Security (PEASEC), Pankratiusstraße 2, 64289 Darmstadt, Germany

**Keywords:** COVID-19, Fake news, Crisis communication, Network analysis, User-centered

## Abstract

In crises such as the COVID-19 pandemic, it is crucial to support users when dealing with social media content. Considering digital resilience, we propose a web app based on Social Network Analysis (SNA) to provide an overview of potentially misleading vs. non-misleading content on Twitter, which can be explored by users and enable foundational learning. The latter aims at systematically identifying thematic patterns which may be associated with misleading information. Additionally, it entails reflecting on indicators of misleading tweets which are proposed to approach classification of tweets. Paying special attention to non-expert users of social media, we conducted a two-step *Think Aloud* study for evaluation. While participants valued the opportunity to generate new knowledge and the diversity of the application, qualities such as equality and rapidity may be further improved. However, learning effects outweighed individual costs as all users were able to shift focus onto relevant features, such as hashtags, while readily pointing out content characteristics. Our design artifact connects to learning-oriented interventions regarding the spread of misleading information and tackles information overload by a SNA-based plug-in.

## Introduction

The COVID-19 pandemic has confronted people all over the world with different challenges. The main challenge concerns measures to contain the virus, which include a variety of government-led measures aiming at physical distancing and vaccination (Desvars-Larrive et al., 2020). Particularly in the context of public health crises, adequate communication of relevant information is crucial. Believing and trusting inaccurate information may not only result in regressive world views but also careless behavior and physical harm of individuals (Guardian, 2020). Considering the spread of misleading information on social media, the World Health Organization (2020) has referred to the problem as an “infodemic”. From the perspective of *Crisis Informatics*, which is dedicated to information systems in disruptive situations (Soden & Palen, 2018; Starbird & Palen, 2011), increasing digital resilience allows individual, informal actors to better contribute to crisis management. The challenge of misleading information has been identified as a barrier to rely on social media in crises (Reuter et al., 2017). While misinformation has been perceived as an exogenous issue, more recent works interested in successful collaboration of social media users have noted ideological foundations and (state) actors’ involvement (Nied et al., 2017; Starbird, 2017; Starbird et al., 2019).

Our work focuses on targeted social media users and proposes to productively encounter misleading information on social media in a context of high uncertainty, need for transparency and higher risk of information overload (Fromm et al., 2021; Kaufhold et al., 2020). Thereby, we assume users with relatively strong media literacy as well as non-expert users to interact with information on social media which is reliable to varying degrees (Kahne & Bowyer, 2017). Looking at empowering ways of assisting users in interacting critically (Mihailidis & Viotty, 2017; Comes et al., 2017), we have developed a prototype of a web app which relies on network analysis. Aware of its relative complexity (García-Saiz et al., 2014), we presume visualization of networks of tweets to offer a usable overview for people with different media competences and prior knowledge. The tool allows to further investigate tweets as it includes word clouds and lists with comprehensible indicators for misleading content. Aiming for digital resilience of user “crowds” (Starbird et al., 2014), it is important to assist users who individually show different levels of motivation, routines and skills. While general short attention spans need to be considered, we assume that assisting tools enhance learning and comprehension especially when individuals use them consciously (Pennycook et al., 2021; Pennycook & Rand, 2019). In return for carrying the costs of dedicated time, users may be supported by a relatively transparent web app which aims to train systematic information processing skills. Thus, we are interested in answering the following research questions (RQs):

1. *How can a web app which aims to foster users’ ability of systematic information processing be designed?*
2. *How can user learning be effectively enhanced in the context of an “infodemic”?*

Following other inquiries of design science (Thuan et al., 2019), we are interested in problem-solving. Thus, we focus on both the development RQ1 and evaluation of a web app which is usable independently of individual media literacy (RQ2).

We particularly aim to respond to its usability for lay users during crises, for which digital resilience is necessary to overcome and adapt to external shocks in the long-term, including potential information overload (Heeks & Ospina, 2018). Building upon social media being considered a tool for individuals to successfully navigate stressful situations (Comes et al., 2017), the web app aims to contribute to digital resilience by empowering users to participate in informed decision-making. More specifically, the web app aims to enhance systematic and analytical processing of information instead of heuristic or intuitive thinking (Hong & Kim, 2020; Misra et al., 2020). On Twitter, information quality of posts may be very heterogeneous and thus pose a cognitive burden on social media users. Our aim is to consider a tool which helps to relieve individuals from this burden, counter information avoidance, and enhance critical, systematic thinking. We further understand motivation to be dynamic (Huang et al., 2018); it is thus important that the web app itself does not contribute to information overload but that it offers a satisfactory experience to both expert and non-expert users (Goudalo & Kolski, 2016; Hong & Kim, 2020).

Ultimately, however, our technical solution carries limitations and should rather be perceived as a complementary approach to other educative endeavors of digital literacy (McDougall, 2019). As noted by Gregor and Hevner (2013), design science research contributes to IS research not only by the practical work of developing an artifact but also by offering theoretical insights of different levels of abstraction. Presenting an “exaptation” (Gregor & Hevner, 2013), we rely on existing technology to address a new problem. Communicating our findings (see Section 6), we discuss results on learning about systematic processing of information on social media to contribute to the knowledge base. Referring to an “infodemic”, we extend our field of interest onto contexts which are characterized by a vast amount of disseminated information of different information quality which increases the potential of individual information overload and intuitive, non-analytical thinking.

Following a design research approach, we refer to the conceptualization of resilience in IS research by Heeks and Ospina (2018) and identify transparency as an additional design requirement. We present the development of a web app in JavaScript and Flutter, relying on pre-established Twitter data sets and SNA in Python. Based on a two-step evaluation (*Think Aloud* study), we find that independent from their level of media literacy, participants were able to generate a specific network and explore it with regards to thematic trends and potentially relevant indicators. However, users with specific background knowledge put more emphasis on the assessment of “black-box” classification of misleading vs. valid information. Flexibility proved to be advantageous while participants were unequally overwhelmed by both the network visualization and misinformation on the COVID-19 pandemic as an issue. This highlights the relevance to consider different levels of media literacy as well as motivations.

Our work pays special attention to strengthening digital resilience of human-social media information systems and proposes learning-oriented ICT (Kahne & Bowyer, 2017). The web app may be embedded as a plug-in and can be accessed by an external link. We therefore offer an alternative intervention to top-down, overwhelming warnings directly integrated in the UI, by providing substantial information on tweets to individual users who are ready to invest more time for learning.

In the following, we present related work (see Section 2) which problematizes user-centered measures against misleading information with regard to users’ systematic information processing in times of crisis. Subsequently, we introduce our *design science approach* (see Section 3) which consists of the formulation of design requirements of a relevant artifact, its development, and an iterative evaluation. We then present the concept and implementation of the web app (see Section 4) and illustrate the main findings of the two-step evaluation (see Section 5). This is followed by a discussion of our results (see Section 6) and complemented by a conclusion (see Section 7).

## Related Work

### Sense-making, Misleading Information and Information Overload in Crises

In disruptive situations, people face a high level of uncertainty as well as the need to exchange necessary information (Dailey & Starbird, 2015; Krause et al., 2020). Processes of sense-making and coping often entail the dissemination of unverified and misleading information on social media (Leavitt & Clark, 2014; Leavitt & Robinson, 2017; Dailey & Starbird, 2014). Leavitt and Robinson (2017) stress that information visibility, afforded by design, is a crucial factor in users’ perception of a crisis on which crowd-based efforts of volunteering, sense-making or emotional support are built on. Although misleading information may be a general problem, its distribution in the context of a global pandemic carries great costs. Science denialism, information overload, avoidance or fatigue may result in the loss of lives due to non-systematic processing or unawareness of relevant information (Hansson, 2017; Hong & Kim, 2020).

While validation of information in the context of crises such as earthquakes may be essential (Goggins et al., 2012), social media users rely on information of different quality and also have to cope with uncertainty in long-term disruptive situations such as epidemics (Sell et al., 2020; Nerghes et al., 2018). Referring to the MERS outbreak in South Korea in 2015, Yang and Lee (2020) emphasize the existence of an “information vacuum” which allows “risk-alarming frames” of media coverage to build on anxiety. As studies on social media use during the COVID-19 pandemic have indicated (Jo et al., 2020; Ahmad & Murad, 2020), anxiety is quite common across the globe. However, responses to anxious posts also comprise misleading information (Ahmad & Murad, 2020) whose display without information on reliability inhibits users from productive interactions. Thus, sense-making in crises, which entails systematic processing of information, takes place in an environment where users more easily perceive information overload and information quality is not necessarily assured (Kaufhold et al., 2020). Thereby, information quality depends on uncertainty, novelty, ambiguity, and complexity (Hong & Kim, 2020). Information accuracy is an important criterion for information quality (Lee et al., 2002) and, following Kahn et al. (2002), Hosseini et al. (2017) relate to different dimensions of information quality, including understandability, when they assess relevant requirements of transparent systems.

As found out by Hong and Kim (2020), information overload, defined as “the state of lacking cognitive resources to process information”, prevents individuals from systematic processing of information, learning, or critical thinking. Instead, people engage in heuristic processing or intuitive thinking, which relies on trusting external opinions and precludes understanding (Misra et al., 2020; Hong & Kim, 2020). Although this may be part of coping with crisis situations, it prohibits users to fully adapt to new contexts. Further, Misra et al. (2020) emphasize that information overload is a subjective experience, which is prevalent when “feeling burdened by large amounts of information” which cannot be used efficiently or effectively. This may be the case in emotional crisis situations (Huang et al., 2015).

### User Media Literacy and Resilient Information Systems

Successful, systematic processing of information relies on a resilient human-social media IS and thus a clear, transparent display of information. It is also positively associated with social media users’ motivation and their levels of media literacy (Hong & Kim, 2020; Koltay, 2012). The broad concept of media literacy allows to capture not only “an individual’s ability to understand printed text and to communicate through print” (Koltay, 2012) but refers to knowledge about different media environments, including (lack of) journalistic verification processes and different forms of representation of information and their purposes (McDougall, 2019). Importantly, McDougall (2019) points out that media literacy is not equal to digital skills but presumes individuals to assess content on topics they are not familiar with based on their critical thinking skills and understanding of media content. Arguing against simplifying solutionist approaches, tools may refrain from suggesting easy, binary categorization into fake news and the truth but instead, aim to enhance social media users’ media literacy and thus processes of analytical or systematic thinking (McDougall, 2019).

Various studies have focused on digital resilience (Garista & Pocetta, 2014) and understand resilience as the “capacity of individuals and communities to bounce back” (Roberts et al., 2015) or even ‘bounce forward’, adapting to external shocks (Müller et al., 2013). Depending on information technology as well as human dispositions, an information system can be improved purposefully and share a diverse set of attributes which reflect resilience, such as learning, rapidity, flexibility & diversity, and equality (Heeks & Ospina, 2018). Considering these attributes, learning is assumed to be foundational (Heeks & Ospina, 2018). As highlighted by other scholars, it implies users’ active engagement which initiates critical thinking (McDougall, 2019; Lehman & Miller, 2020). Resilient information systems do not exclude the potential of information overload a priori. Instead, they allow for learning from related experiences, i.e., increasing media literacy (McDougall, 2019), and to foster systematic information processing over heuristic thinking.

While Koltay (2012) as well as Hong and Kim (2020) found media literacy to be a more important factor influencing the potential of information overload, users’ socio-economic background may play a role as well (Seaborn et al., 2021). Considering user modelling for enhanced requirement specification, Adikari et al. (2006) point out that users are generally assumed to be able-bodied and may have different needs, goals, and preferences. As with information overload, user experience is also subjective (Goudalo & Kolski, 2016; Vermeeren et al., 2010). Besides media literacy, Hong and Kim (2020) identify motivation to be essential for learning. However, affected by user experience and changing goals, motivation of both “professionals and amateurs” (Koltay, 2012) is a dynamic variable (Vermeeren et al., 2010; Huang et al., 2018). Motivational factors are an individual’s attitude and emotional state, entertainment gratification as well as perceived ease of use (Huang et al., 2018). The latter may be defined by the extent of old knowledge that could be used to routinely interact with information technology (Naumann et al., 2007). However, McDougall (2019) emphasizes that while critical media literacy is more likely to foster digital resilience, it should not be framed as an individual responsibility.

### Suitability of Existing Approaches

To avoid information overload for social media users, automatic detection efforts have been made (Wu & Liu, 2018; Zhou & Zafarani, 2020). However, accurate detection may be challenging. For example, Shu et al. (2017) point out the topical diversity of misleading content and the increasing importance of rhetorical structures and Zhou and Zafarani (2020) capture the relevance of different categorization of content. Besides diverse approaches of detection, technical interventions may be beneficial when they differentiate between “malicious” and “normal” users, by assisting the latter to “improve their ability to distinguish fake news” (Zhou & Zafarani, 2020).

In this regard, Clayton et al. (2020) find that general warnings about media were even counterproductive while more specific warnings show a small positive effect. Further, their study shows that “rated false” tags are more helpful in correcting views on information accuracy than displays of posts being “disputed”.

Following inoculation theory, Roozenbeek and van der Linden (2018) analyze the effect of an online “serious” game, in which users play the role of creators of misinformation. Although isolated from real-world social media environments, a learning game emphasizes the importance of substantial interventions (Roozenbeek & van der Linden, 2018). Further, Guess et al. (2020) emphasize that discernment between false and accurate news can be increased by “teaching people more effective heuristics” instead of relying on quicker, cost-sensitive measures (Thirumuruganathan et al., 2021) which require users to pay attention to the assessment of singular posts. Lehman and Miller (2020) also note that active engagement (in their case microblogging) increases learning effects of individuals. In case users find interaction “simple, fun and educational” (Micallef et al., 2021), they are ready to invest time and increase their media literacy.

In sum, the relevance of learning with regard to interacting with misleading information autonomously is highlighted by different studies, emphasizing the enhancement of media literacy as promising (de Cock Buning, 2018; Kahne & Bowyer, 2017; Mihailidis & Viotty, 2017). Using Agent-Based Modelling, Gausen et al. (2021) evaluate measures against misinformation, distinguishing between blocking users, as well as behavioral approaches, accuracy flags, and “societal inoculation” (Lewandowsky & van der Linden, 2021). The latter implies that it is necessary to create “mental antibodies” (Goldberg, 2021) against misinformation by fostering users’ skills to assess information accurately.

As related scholarly work on the COVID-19 pandemic has indicated, Social Network Analysis (SNA) is assumed to produce generalizable insights into the distribution of content on social media. Thus, it offers grounds on which users can build insights into broader debates and learn about narratives. For example, Ahmed et al. (2020) use SNA and present clusters which reflect debates surrounding 5G and COVID-19, including relevant actors on social media. Similarly, Memon and Carley (2020) focus on propagating actors of COVID-19-related misinformation, presenting insights regarding misinformed communities on Twitter. Milani et al. (2020) also analyze diffusion networks regarding vaccination debates on social media which focus on polarizing actors. Their model has been implemented by Cui et al. (2019). It assesses content for interested users and allows them to track a post’s dissemination using a network graph. While Shao et al. (2018) propose Hoaxy which allows to gain insight into the diffusion of misinformation by studying user networks, Yan et al. (2012) also include visualized topical groups of tweets based on their semantic proximity. Proposing Check-It, Paschalides et al. (2019) combine user analysis of online social networks with a Deep Neural Network for linguistic analysis and cross-checking articles with a list of fact-checking websites. Sharma et al. (2020) investigated narratives that were promoted in misleading tweets in English during the COVID-19 crisis in 182 countries. It must be taken into account, that as pointed out by Shu et al. (2019), nodes of networks do not necessarily reflect users.

Further, the display of comprehensible indicators, giving insight into potential criteria for classification, may reduce reactant behavior by social media users as countermeasures are no longer perceived as top-down initiatives (Richards & Banas, 2015). For example, Aswani et al. (2019) and Hartwig and Reuter (2019) propose understandable indicators for misleading social media content. Such “white-box” approaches allow users to get an impression of what happens during information processing between in- and output. Results of a survey conducted by Kirchner and Reuter (2020) support the need for comprehensible countermeasures in contrast to non-transparent warning signs, with respondents demanding explanations. However, transparency itself can lead to information overload in the absence of information accuracy, clarity, accessibility, or understandability (Hosseini et al., 2017). Therefore, the importance of information quality should not be neglected in transparent information systems.

Various works capture the diverse technical countermeasures both proposed and undertaken by research and social media platforms (Courchesne et al., 2021; Yadav, 2021; Gausen et al., 2021). Roughly, it is differentiated between deletion, redirection or reduction of visibility of posts, and behavioral approaches tackling users’ perception of social media content by warnings or labeling. Looking at existing approaches, there is little focus on introducing surrounding broader debates or giving more specific explanations to labeled content independent from user features (see Table 1).

**Table 1:** Examples of existing countermeasures implemented in major social media platforms; own research

Social Media	Counter-	Binary	Learning	Top-down	Broader
	measures	Labels	Effect	Bottom-up	Debate
Twitter (2020)	labels	no	yes (explained by user features, further info)	top-down	no
	warnings	yes	yes (explained by user features, further info)	top-down	no
	reduced visibility	n/a	no	top-down	no
Birdwatch (Twitter, 2021)	labels	no	yes (explained by thematic features, further info)	bottom-up	yes
Facebook (Meta, 2022)	labels	no	yes (explained by user features, partly further info)	collab.	no
	warnings	yes	yes (explained by user features, partly further info)	collab.	no
	reduced visibility	n/a	no	top-down	no
Instagram (2019)	labels	no	yes (further info)	collab.	no
	warnings	yes	yes (further info)	collab.	no
	reduced visibility	n/a	no	top-down	no
YouTube (CNBC, 2021)	labels (on spec. topics)	yes	yes (explained by user features, further info)	top-down	no
	reduced visibility	n/a	no	top-down	no
Reddit (CNN, 2021)	bans, “qua-rantine” of subreddits	yes	no	collab.	no
Tik Tok (SMT, 2021)	label	no	yes (further information)	collab.	no

Considering prior findings on media literacy, information overload in crises, and resilience as well as currently implemented countermeasures on social media, we aim for the development of a web app which efficiently enhances users’ systematic processing of social media content. Thus, we follow technical approaches which focus on“societal inoculation” (Gausen et al., 2021; Goldberg, 2021) against misinformation and aim at reducing information overload and fatigue in the context of a pandemic. Aimed at attributes of resilient information systems (Heeks & Ospina, 2018) and transparency implying information quality (Hosseini et al., 2017), the proposed intervention invites users to explore thematic patterns of misleading information and specifically considers the costly challenge of information overload.

## Methodology

Considering misleading communication on social media regarding the COVID-19 pandemic and platforms’ rather reserved interventions, we find the design and development of a web application which supports long-term adaptation and transformation of problematic phenomena highly relevant. Thus, we conducted a *design science approach* as an appropriate method to create new and innovative artifacts. Based on an identified empirical problem, this approach comprises the design of an artifact as well as an evaluation, aimed at systematic problem-solving (Hevner et al., 2004). Contributing to IS research with an exaptation (Gregor & Hevner, 2013), we subsequently communicate more abstract findings which can be derived from the instantiated artifact. Connecting to the existing knowledge base on misinformation, information processing and overload as well as digitally resilient information systems, formulated implications refer to both kernel theories about human behavior and mid range theories on design and crisis management (Gregor & Hevner, 2013). While we do not contribute with insights on the third level of abstraction, i.e., fully developed design theories, the situated implementation (first level) is a contribution in itself and also allows for the discussion of rules with regard to the design of Internet-related socio-technical information systems (second level) (Gregor & Hevner, 2013).

In our case, the artifact is a web app and evaluations took place iteratively in form of two *Think Aloud* studies. The design science approach consists of five steps, namely (a) achieving problem awareness and showing its importance, (b) suggesting a solution’s objectives, (c) the web app’s design and development as a solution to the problem in a suitable context, (d) an iterative evaluation of the artifact using the *Think Aloud* method, and finally (e) the communication of a conclusion (Pfeffers et al., 2006).

We follow an iterative evaluation process in which a two-step *Think Aloud* study aims at refining the design of the web app (Hevner et al., 2004). *Think Aloud* studies are a suitable method to gain rich qualitative and user-centered feedback on problem-solving, using a novel artifact such as our web app. Performing the two studies iteratively has facilitated the refinement of the artifact based on obtained findings (Fonteyn et al., 1993).

While specific empirical analyses may precede the development of applications (Reuter et al., 2014; Wulf et al., 2011), our work follows existing studies which have noted the importance of social media for coping with crises and identified related problems of unreliable content and information overload (Comes et al., 2017; Fromm et al., 2021; Tran et al., 2020). We also rely on insights of studies on the spread of misinformation, particularly noting users’ needs for an explanation as well as the unsustainability of low-effort interventions (Roozenbeek & van der Linden, 2018; Kirchner & Reuter, 2020; Pennycook et al., 2021). Considering disruptive situations in which individuals need to manage external shocks, we are specifically interested in user-assisting tools which enable individuals to learn. Linking the question of strengthening digital resilience to media literacy as a basis for handling crises allows focusing on identified attributes of the former (Heeks & Ospina, 2018). These help to structure both the development process and evaluation. Thus, our perspective allows to combine efforts of developing problem-solving applications with the generation of insights complementing the IS knowledge base (Hevner et al., 2004). In the following, we present the concept and implementation of the web app (see Section 4) and findings of the two-step evaluation (see Section  5.3) which point out both potentials and limitations determining the process of learning, i.e., increasing digital resilience.

## A Concept for Digital Resilience on Social Media

We propose a web app to provide social media users with an overview of potentially misleading and non-misleading tweets with the aim to encourage media literacy and thus improved digital resilience and also investigated how the latter can be increased using an interactive tool based on SNA. We used the four attributes of *learning*, *diversity & flexibility*, *equality*, and *rapidity*, characterizing digital resilience (Heeks & Ospina, 2018) as a frame of reference for the development process. Derived from the knowledge base (see Section 2), we present *transparency* as an additional design requirement (see Table 2).

Assuming that users can benefit from an intervention which considers specific posts but emphasizes their thematic connections, the SNA-based web app shifts the focus away from singular instances of misleading information onto topical trends which updated are more (or less) prevalent in misleading or valid tweets. We chose this approach to offer a user-assisting tool which enables individuals’ to improve their systematic processing skills and discernment of posts’ information quality in the context of crisis situations.

Despite varying attention spans and potential information overload, users are encouraged to actively generate network visualizations and focus on classifying information as valid or misleading. Thus, the web app can be considered a tool to enhance crucial learning processes. While this engagement takes time, we aim for a relatively easy process of network generation, highlighting the function while further exploration of tweets and proposed indicators are added to an information system which allows diversity & flexibility. Aware of different user backgrounds, i.e., personal motivation and media literacy, it is crucial to offer various options to generate resources. Especially during the COVID-19 pandemic, which has accelerated digitization, it is relevant to include new users and allow digital resilience to be fostered independent of their level of media literacy.

**Table 2:** Design requirements contributing to digital resilience. Interested in increasing media literacy, we rely on resilience attributes and identified indicators by Heeks and Ospina (2018) which imply learning. Transparency was added as another abstract design requirement during initial awareness raising. Thereby, we used findings by related works (see Section 2) which also involved considering *user attributes*

Knowledge Base	Requirements	Indicators
– *user needs: sensemaking* – low info quality: high uncertainty, novel, ambiguous, complex contents in crisis – info visibility is crucial – info quality, including accuracy is necessary in transparency rhetorical structure plays a role	Transparency	accessibility, consistency, certainty, accuracy, understandability, clarity
– training media literacy: systematic instead of heuristic processing – understanding processes of information production based on printed text – reduction of info overload – understanding broader narratives, topical diversity – active involvement – generalizable results on which users can build insights	Learning	capacity-building, new & trad. knowledge, reflective thinking
*different needs, goals, preferences of users*	Diversity & Flexibility	diff. courses of action, emerging opportunities, adaptable decision-making, innovation mechanism
– life-long learning independent from *media literacy and sociodemographic factors* – *expert & non-expert users* – *motivation is dynamic* and should not be decreased	Equality	strengthened competencies, gap reduction, inclusiveness, openness & accountability
– user costs (investing time) – ease of use & entertainment – topic is stressful – *motivation is dynamic* and should not be decreased – info avoidance, fatigue are problematic in crisis	Rapidity	rapid resource access, assessment/coordination, mobilization

The backend of the web app was implemented using *Python 3.9* (2020). The *Flask* library builds an HTTP interface that is used to access the frontend. *NetworkX* enables the generation of relatively large graphs and also offers implementations of graph and network algorithms. Most of the UI is written in *Flutter* (Google, 2020), a framework for *Dart*. The generated network is visualized with the JavaScript library *D3.js* (Data-Driven Documents) version 6 (Bostock, 2020). While the web app was developed using state-of-the-art principles and algorithms (e.g., the CNM algorithm to divide nodes into groups), it comes with potential for the innovative user-centered exploration of a social network in the context of misleading information.

### General Concept

**Fig. 1:**
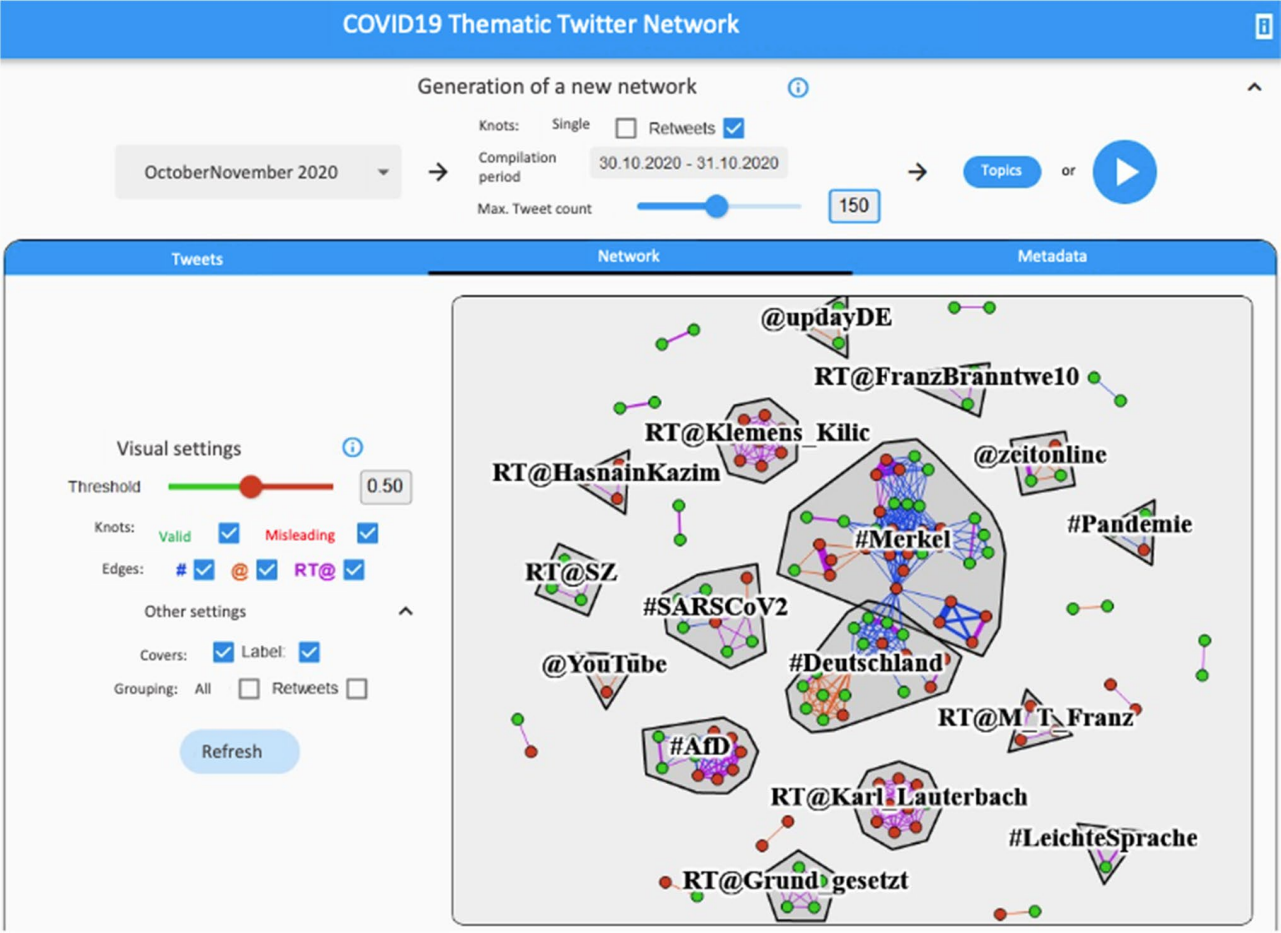
The interface of the web app while the generated network is selected as a visualization component

The web app interface consists of four main components (see Fig. 1).

First, at the top of the interface, there are several options to set up the web app, e.g., by loading a specific data set and making customized configurations before generating a network. Therefore, users’ active participation is required, which in turn encourages learning (McDougall, 2019; Lehman & Miller, 2020; Guess et al., 2020). Second, the core of the web app represents the visualization of a social network for Twitter contents. Third, besides exploring Twitter contents in the visualized social network, the user can switch the data representation towards a list of tweets along with other relevant information. Fourth, the contents of a Twitter data set are visualized as word clouds when selecting the component “metadata”. Offering diverse options for exploration, the web app allows flexibility (Heeks & Ospina, 2018). Users can dive deeper into data and recognize known system features of Twitter, while others preferably rely on the broad thematic overview (Koltay, 2012; Hong & Kim, 2020).

All four components of the interface include options for user configurations regarding the specific representation of data to encourage interactive exploration. In the upper area of the interface, users can generate a new network at any time (see Section 4.2). The lower area either shows a network visualization (see Section 4.3), a list of all used tweets (see Section 4.4), or various metadata as word clouds (see Section 4.5). This ensures the web app can be used by users with different preferences (Adikari et al., 2006). In all areas, tool tips (text that is displayed with a mouse-over effect), provide users with comprehensible descriptions for individual components, thereby contributing to information quality as a requirement for transparency (Hosseini et al., 2017).

### Data Set & Setup

For evaluation purposes, we used predefined data sets (see Section 5.2). Thus, users needed to load a csv-file containing tweets and additional information (separator “;”) to start both generating and exploring a network. However, the web app allows for any further data set to be uploaded, as long as it fits the format. Each row represents one tweet. Information about the tweet is stored as a JSON string (see Table 3).

**Table 3:** Overview of data set

Column	Keys	Description
Tweet	content	raw tweet text
	enrichedData	optional, list of occurring hashtags
	mentions	optional, list of occurring mentions incl. retweet, reply
	retweetTo	Twitter ID of the originating, retweeted tweet
	startTime	posting time, year-month-day-hour:minute:second.msecondZ
	url	URL of the tweet
User	content	optional, raw text of the account description
	isVerified	whether the account is verified
	numStatuses	number of created statuses (tweets)
	numFollower	number of accounts that follow creating account
Score	scoreBERT	optional, decision of BERT classifier (value betw. 0 and 1)

Our data sets for evaluation contain the output of a trained “black-box” classifier to automatically detect misleading contents based on BERT (Bidirectional Encoder Representations from Transformers), a state-of-the-art NLP model from Google (Devlin et al., 2019). Its output is a number between 0 (probably not misleading content) and 1 (probably misleading content). The classifier achieved an accuracy of 0.70 (precision: 0.74, recall: 0.66, F$_{1}$-score: 0.70). BERT calculations were performed beforehand and its outputs were included into the data sets for evaluation, rather than performing the classification during run-time while using the web app.

To explore Twitter contents using the web app, the user first specifies a setup scenario. Therefore, a predefined source data as csv-file is selected containing tweets and associated relevant information (see Section 4.2). Subsequently, users can make additional configurations. For instance, a limited time frame for tweets within the data set can be chosen by selecting specific start and end period for when the tweets were created. This information is stored within the csv-file. Further, users can customize the visualization, by deciding whether to include retweets or by choosing to display isolated nodes without connecting edges. Users further have the possibility to define the maximum amount of tweets to be included in the network. Finally, the hashtags and mentions used to connect nodes can be adapted manually, with which individuals can identify terms that are relevant for a specific narrative (Leavitt & Robinson, 2017; Sharma et al., 2020).

In our scenario, hashtags containing “Corona” or “COVID” are not used as connecting edges by default, as the data collections used for evaluation solely contain tweets related to COVID-19 (see Section 5.2). For all steps of the setup phase, users can access additional information and explanations when moving their mouse over the tool tips. This increases transparency while principally keeping information as concise as possible (Hosseini et al., 2017; Lee et al., 2002). To gain information about German Twitter communication regarding the COVID-19 pandemic, users need to click on the “play”-button, resulting in the calculations for three distinct visualizations (social network, list of tweets, word clouds) of the chosen Twitter contents which all offer different possibilities for interactive exploration.

### Network

**Fig. 2:**
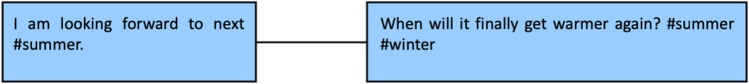
An example of two tweets being connected by hashtags. The authors have been omitted here

Based on the data, a node models a tweet and its author while undirected edges represent thematic connections between the tweets. These are established by hashtags (#<tag>) or mentions (@<username>) (see Fig. 2). A sub-type of mentions used here are “retweets”, i.e., forwardings of (foreign) tweets to one’s own contacts. These are usually marked in the tweet text (“RT @<username>”) and can also be clearly identified by the collected metadata. The type of edge depends on the labels (i.e., hashtags, mentions or retweets) used and the number of connections. A retweet is weighted heavier, since hashtags and mentions are often similar in such cases. As mentions always contain a specific user name, a connection between public figures and specific tweets can be established. To model the topics or trends of the created network, the nodes are divided into groups using the CNM algorithm (Clauset et al., 2004). The most frequent hashtag, mention or retweet, used in the connections is displayed as the entire group’s topic.

While the network visualizes relations which do not represent concrete behavior between user accounts, it shifts focus away from personalized interactions onto relevant topics (Shu et al., 2017, 2019; Yan et al., 2012). This builds grounds for enhancing systematic processing of users as they gain more general insights into the dynamic debate (Shu et al., 2017). Prioritizing edges (mentions, hashtags, retweets) over nodes reflects the relevance of these system features in affording “information visibility” (Leavitt & Robinson, 2017). Thus, individuals can learn about dynamics and functions of social media which fosters critical media literacy. Network visualization of Twitter communication on the COVID-19 pandemic allows to gain a broad overview. It thereby does not only allow to identify thematic patterns but also to relieve users from anxiety during emotional encounters (Yang & Lee, 2020; Jo et al., 2020; Ahmad & Murad, 2020).

The visualization of the network is the core of the web app (see Fig. 1). A “classic” representation is used, i.e., nodes are modeled by circles and edges with lines between circles, which are also colored differently according to the available properties, for example “misleading” or “valid” for nodes or “hashtag”, “mention”, or “retweet” for edges. The visualization can also be configured by the users, for example by hiding certain nodes or edges. The predominant topic (= label) of a group of nodes is displayed in its center.

Within the network component, further configurations can flexibly be made while exploring the data (see Fig. 1).

As a focus of our work is to encourage user exploration of Twitter contents regarding misleading versus non-misleading tweets, the output of the BERT-classification of tweets as probably misleading or not misleading is displayed within the network component. To enhance autonomy for advanced users, users can adapt the threshold which adjusts from which score a tweet should be displayed as “misleading”. This value can be set at any time, so there is no static binary classification. However, the threshold is set to 0.5 by default, while “valid” nodes are colored green and “misleading” nodes are colored red to present a simplified dichotomous differentiation for lay users in a situation where information quality is low due to its novelty and complexity (Hong & Kim, 2020).

A slider or input box can be used to set the threshold for misleading tweets. This ensures users are aware they are dealing with probabilities which technical solutions have to offer and more easily invest time to engage (Hosseini et al., 2017; McDougall, 2019; Roozenbeek & van der Linden, 2018; Thirumuruganathan et al., 2021).

Several check boxes can be used to hide certain nodes (misleading, not misleading) or edges (hashtag, mention, retweet) depending on user preferences (Adikari et al., 2006). Related to the grouping, borders or topic labels can be drawn or, for further abstraction, all related tweets can be reduced to one node. The labels represent the most popular labels of the respective group (see Fig. 1, right). If the network contains multiple retweets of a particular tweet, these can also be reduced to one node. The web app displays the network graph interactively. Users can drag individual nodes across the canvas by holding down the left mouse button, or single left-click to explore selected tweet data in a new window. When the mouse hovers over a node or edge, some data is displayed in a tool tip, ensuring accuracy and accessibility of information (Hosseini et al., 2017). The mouse wheel enlarges the display and allows a more detailed view of the network.

Depending on the settings above, nodes and edges are colored in certain colors. If preferred, an alternative color scheme according to Wong (2011) is used. The thickness of the edges shows the number of connections between two tweets. When tweets are reduced to groups, the display slightly changes. The size of the nodes (= groups) then represents the number of attributed tweets; analogously the thickness of the edges symbolizes the number of connections between two groups. The score of a group is the median of all associated tweet scores. A left mouse click on a node displays all tweets and some metadata of the selected group in the new window.

### Tweets

In addition to the network component, the web app offers an exploration of Twitter contents by displaying all tweets as a list along with relevant information. In contrast to the network graph, the display is largely text-based but also allows a certain degree of filtering or sorting. Thereby, one can take a closer look at the tweets used in the network. All relevant node data is displayed for each tweet. If preferred, the tweet can be opened in a new window. In addition, the list can be further customized: A slider sets a minimum and a maximum score of the BERT classifier, retweets can be hidden and a drop-down menu allows the selection of a sorting order. If preferred, the value can be displayed as a percentage instead of the decimal score. In addition to the BERT classification, we detect and display comprehensible indicators for misleading information during runtime within this component; having been inspired by prior research:

1. capitalization (Wanas et al., 2008; Hartwig & Reuter, 2019)
2. frequent punctuation marks (Wanas et al., 2008; Hartwig & Reuter, 2019)
3. frequent and conspicuous emoticons (Morris et al. 2012; Hartwig & Reuter, 2019)
4. frequent hashtags (Aswani et al., 2019)
5. number of URLs (Castillo et al., 2011)

While such attributes do not offer a holistic explanation for contents’ misleading character and are by far not deterministic in this regard, indicators function as points of references for systematic processing of information which is crucial for users (Kirchner & Reuter, 2020; Hong & Kim, 2020). Critical assessment of tweets includes considering highly emotional content and relying on bridging system features, such as hashtags or embedded links (Huang et al., 2015; Leavitt & Robinson, 2017). To limit the potential of suggesting indicators to adequately explain BERT scores, we refrain from causal statements and opt for clear and consistent (Lee et al., 2002) display of indicators independent from score values. While indicators are generally comprehensible, engaging users rely on their prior knowledge about social media features at the beginning (Hosseini et al., 2017).

### Metadata

The last visualization component displays all detected labels (separated by hashtags and mentions) in word clouds and the labels that occur together are listed. The metadata area component displays various information about the generated network. The size of the text depends on the absolute frequency of the label. Labels that were used only in one tweet or that directly contain “Corona” are also displayed using a checkbox. Further below, connections between labels are shown. Display of connections between topical labels as well as word clouds pose the most abstract and simplified exploration of Twitter data as there is no corresponding presentation by tweets or visualization of thematic relationships. For reproduction purposes, the parameters used in the last setup phase can be viewed in this area.

## Evaluation

In the following, we present our method (see Section 5.1) as well as the pre-established data sets used for the evaluation (see Section 5.2). Subsequently, relevant findings considering digital resilience are illustrated (see Section 5.3).

### Method

The web app was evaluated in a two-step *Think Aloud* study (Fonteyn et al., 1993). Prior to this, a test run was also carried out with four people which allowed the test leader to prepare and check the recording procedure (Hegner, 2003). Responding to the feedback, a search box for tweets was added. The iterative approach was guided by the aim of improving the web app, paying special attention to the needs of non-expert users. Both evaluative meetings took place on Zoom with 16 participants. Nine people participated in the first round between February 27, 2021 and March 1, 2021 and seven participated in the second evaluation between March 8, 2021 and March 11, 2021. On average, each unit lasted 39:14 minutes or 42:36 minutes, respectively. To avoid bias by prior experience, subjects participated only once in total. Individuals differed in age and motivation. We further distinguished between lay users and experts according to level of media literacy. This in turn is understood as dependent on habitual Twitter use and media consumption (see App. Table 6).

The participants were briefed on the *Think Aloud* method (Fonteyn et al., 1993) and consented to their session being recorded for the purposes of this scientific study. Further, a friendly and relaxed atmosphere was critical to create a quiet space in which participants could articulate their thoughts freely (Fonteyn et al., 1993). The undertaking researcher thus used appropriate language and clarified that participants’ thoughts were not categorized as right or wrong. He mostly refrained from interfering to not interrupt participants but promoted the process of “thinking aloud” by asking open questions (see App. Table 7). Since the test persons usually have little or no prior knowledge in the field of networks, an introduction (see App., Fig. 7) was handed out and gone through after an initial phase of exploration. Thereby, it was not only ensured that participants had a“general impression of the system” (Hegner, 2003) but also indicated differences in use, based on individual background.

As part of the design product evaluation (Shrestha et al., 2014), a prefabricated task was presented subsequently, which was carried out by each participant in interaction with the web app (see App., Table 7, 3.). This comprised a guided network creation, identifying a predominant topic, and searching for indicators of misleading information in tweets with “#Merkel”. Simultaneously, participants were encouraged to express their thoughts out loud. The researcher also took additional notes regarding possible ambiguities or peculiarities. The transcript and the standardized tasks allow for comparison of usage. Following Fonteyn et al. (1993), a conventional interview phase was conducted succeeding the *Think Aloud* phase. The latter allows a direct, consistent insight into participants’ thoughts, while the former indicates their retrospective views. Following the meetings, the researcher transcribed the audio material from which we then derived findings regarding digital resilience.

### Test Data Set

For the evaluation, users relied on prior collected data sets of three observation periods (see Table 4). Therefore, in each of the time periods, the query “Coronavirus OR Corona OR Covid OR Coronakrise OR coronadeutschland OR Corona19 OR covid19 OR covid-19 OR #COVID19” was sent to a Twitter API. The data sets were further restricted to tweets in the German language (“language = de”) and thus reduced in size. Limiting text length of retweets to 140 characters, the data files also include respective tweets but may not comprise the entire post. Afterwards, for each tweet a pre-trained BERT classifier calculated a score between 0 and 1 which indicates the accordance with misleading contents (see Section 4.2). The filtered data, complemented by the BERT-output, was then saved in a specific format (see Section 4.2).

**Table 4:** Collection of test data sets. Observation spanned over diverse phases of the long-term crisis to capture Twitter communication in the context of different numbers of infected cases and governmental restrictions

Observation Period	Tweets	Crisis Context in Germany
02/27/2020-03/06/2020	156,455	beginning of crisis (infections, public debate)
04/14/2020-04/22/2020	205,198	during first “shut-down”, gov. restrictions
10/29/2020-11/27/2020	334,215	during “lock-down light”, before second one

### Results

Aiming for digital resilience, we structured the analysis of the two sessions according to formulated design requirements (see Table 2). Our work is a proposal and offers ground for identification of both potentials and limitations of resilient user-assisting technology.

#### Transparency

Considering attributes of resilience (Heeks & Ospina, 2018), in particular the essential characteristic of *learning*, these may only be prevalent when technical systems are transparent to users and when *transparency* also implies information quality (Hosseini et al., 2017). Accessing the application, some participants (e.g., EVAL-1A) wished for a better introduction. In particular, this was more relevant to individuals who had had less prior engagement into networks (EVAL-1C). This was contrasted by one user who understood the purpose of the network visualization immediately: “*As I have already worked on own data sets and built edges and nodes [...], it is a bit easier for me to understand*” (EVAL-1B). Thereby, they could rely on prior knowledge (Heeks & Ospina, 2018), while other users could only access new knowledge after an expanded introduction to the web app as well as a continuous text description had been provided for overall comprehension (Hosseini et al., 2017).

Further, users who were relatively new to social media communication did not change the threshold and adjust probabilities of classifying content as misleading or valid, taking BERT results, when asked explicitly, as “*given*” (EVAL-1C). Only expert users showed awareness regarding the slider and were eager to gain more detailed insight. Although tool tips were often disregarded in both the first and second evaluation, individuals who showed higher interest and felt comfortable with exploring the application could get more insights which was found highly relevant in terms of their perception on the classification of content (EVAL-1A). At the same time, binary coloring supported users who did not dedicate their time to exploring the threshold. This shows the importance of transparency to users’ experiences while effectiveness of design depends on their media literacy and information quality, indicating a subjective notion of understandability (Hosseini et al., 2017).

#### Learning

Learning is foundational to digital resilience as it builds ground for adapting to disruptive situations (Heeks & Ospina, 2018). Considering both expert and lay users, we evaluated the web app’s usage regarding the potential for capacity-building, opportunities to rely on (new and old) knowledge, as well as systematic, reflective thinking (Heeks & Ospina, 2018; Hong & Kim, 2020; McDougall, 2019). Presenting networks to users, we aimed at offering individuals with insights into thematic connections between tweets which may be more (or less) prevalent among misleading (or non-misleading) posts. In this regard, all participants were able to acquire new knowledge while all of them were informed, albeit to varying degrees, about the COVID-19 pandemic and aware of the spread of misinformation. For example, EVAL-1B, who mainly relies on conventional media for news consumption and is not active on Twitter, concluded that

“*one may assess clearly that, here, clusters are established comprising valid and some misleading [tweets; AN] and that there are also sometimes connections between accurate and misleading information [...].*“

Another participant who does not use social media and felt a bit overwhelmed in the beginning could subsequently identify thematic patterns such as on politicians of the German government (EVAL-2L, see App., Figs. 8, 9). This indicates that although individuals may not habitually use social media, overview of Twitter communication by networks allows for systematic processing of information (Hong & Kim, 2020). In particular, description of thematic communities and notification of connections among tweets of varying accuracy reflect active engagement and critical reflection instead of intuitive thinking (Guess et al., 2020; Heeks & Ospina, 2018; Misra et al., 2020). However, only expert users (EVAL-1I, EVAL-2M) followed the idea of a misleading-valid continuum (McDougall, 2019) and were open to depart from perceiving misinformation as a clearly and easily identifiable issue. They showed awareness regarding the slider and were eager to gain more detailed insight. They also used the tweet tab for better understanding (EVAL-2M). Changed prior to the second evaluation, binary coloring of the slider supported heuristic thinking (Hong & Kim, 2020) by lay users, connecting the threshold to the network:

“*Uh, it says ‘valid’ and ‘misleading’. So red is misleading and, uh, to some degree fake or at least you don’t know whether it is true. And when colored green, you may believe it is true [...]* (EVAL-2P).

Participants also started to evaluate proposed indicators of misleading tweets and autonomously weighted them against user biographies or linguistic content (EVAL-1H, EVAL-2K). Learning to differentiate between misleading and valid tweets was also initiated by individuals who did not focus by themselves on proposed indicators and had less background knowledge about Twitter, pointing out “*dubious terms*”, a user’s biography and grammatical inconsistencies (EVAL-2J, EVAL-2L). Thus, although active generation of a network proved to be difficult to some subjects, they could expand prior knowledge and gain new insights into potential indicators of misleading information. As such heuristic cues (Hong & Kim, 2020) were already presented to users, they could train systematic processing of information and gain an understanding on how narratives on social media are developed (Leavitt & Robinson, 2017).

#### Rapidity

In the context of varying attention spans and quick interactions on social media (Pennycook et al., 2021), resilient systems are perceived to provide rapid access to resources, i.e. information, and allow for their efficient assessment, coordination and mobilization (Heeks & Ospina, 2018; Goudalo & Kolski, 2016). In this regard, initial interaction with the web app is crucial which was at times perceived to require too much effort. One user pointed out that one could familiarize oneself with the application within “*5 minutes* and indicated the need for improvement by including a “*30-seconds video*” or a “*short explanatory text*” (EVAL-1E). Thus, we integrated the latter prior to the second evaluation to accelerate the learning process. Generating a network based on selecting a data set and time frame provided some difficulties to lay users. In general, participants needed several minutes to fulfill the task of generating a network. The time required by participants to fulfill this task was reduced by changes to the generation UI between the first and second evaluation (see Table 5).

**Table 5:** Duration of a specific network generation in minutes. The median value of the second evaluation is lower than of the first experiment. One value (8:55 min) had to be corrected because of an interruption of 15 minutes without internet access

EVAL-1A-1I	4:43	5:00	5:56	7:13	1:56	7:03	3:44	3:22	4:53
EVAL-2J-2P	2:28	3:20	5:54	3:05	3:08	8:55	10:24		

In terms of individual times, it should be considered that participants were asked to fulfill a standardized task of generating a specific network. This was done to gain more systematic, yet non-representative, results. Certainly, this took more time than passively noting warning signs. However, once they started, participants paid attention and continued with the task, simultaneously articulating their thoughts. As the start, i.e., selection of the data set and time frame as well as labels required at times more effort than anticipated (EVAL-2M), respective improvement is crucial to increase digital resilience.

**Fig. 3:**
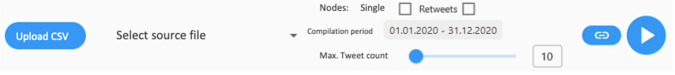
Evaluation 1: If, for example, the play button is pressed without a selected record, a new window appears with a note

**Fig. 4:**
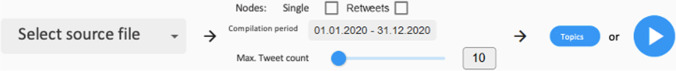
Evaluation 2: Instead of a popup, the drop-down box is colored yellow and a snack bar with a hint is displayed. Arrows reflect the procedural steps for network generation while terms and icons which were perceived irritating in the first evaluation have been eliminated or changed

Based on the first evaluation and the experiment’s frame of using predefined data sets, users found the “Upload CSV” button off-putting (EVAL-1E). To ensure user-friendliness, CVS.files can now be uploaded by accessing the drop down menu and selecting “select source file” (see Figs. 3 and 4). Further, we included two arrows to illustrate the process and while a pop-up window appeared in the first evaluation, which some perceived as overwhelming (EVAL-1A), the drop-down field is colored yellow and a hint to select a file is presented with an automatically disappearing snack bar at the bottom of the screen. The first evaluation indicated that the steps necessary for successful network creation were not clear, with users showing frustration over attempts to select time periods (but no data set) (EVAL-1E). Transparency, characterized by information quality, is thus crucial for efficient network creation and to avoid information overload (Hosseini et al., 2017; Lee et al., 2002; Hong & Kim, 2020; Misra et al., 2020). Additionally, the icon representing a chain of connections was replaced by text (“Topics”) as it was misunderstood prior the second evaluation (EVAL-1D). While participants during the second evaluation neither articulated positive feedback regarding the “topics” button, nor explicitly focused on it, it did not inhibit network creation. EVAL-2J was, however, able to describe its function retrospectively: “*Well, I can’t remember what the window was, but it probably selects individual clusters in the data set*”.

These changes are particularly relevant to avoid future decreases in motivation as readiness to engage with social media content was sometimes low with subjects being, as expected (Jo et al., 2020; Ahmad & Murad, 2020; Hong & Kim, 2020), “*annoyed*” (EVAL-2M) or “*frustrated*” (EVAL-1I) by the pandemic.

#### Diversity & Flexibility

Complementing network visualizations, the web app offers a display of tweet contents which may be more known to users. Thereby, users valued that they can gain more detailed insights into tweets which are part of the generated network (EVAL-1I). Further, some participants (e.g. EVAL-2N) autonomously focused on the word cloud when exploring Twitter communication on the COVID-19 pandemic which emphasizes the importance of an information system’s ability to offer various options (Heeks & Ospina, 2018). While the network component is certainly at the core of our approach, it is relevant to offer additional information to users which may not be satisfied by it. EVAL-2J highlighted that they

“*definitely [liked] the way of looking at these graphs, meaning the changes one can make to the network visualization. At the same time, [I like] that one can have a closer look right and left at the metadata and tweets. I assume this [the network, AN] is the main function here and I think that’s indeed well done.*”

Thus, we offer a more traditional display of lists of tweets which also allows users to look at user characteristics such as the bio and thereby rely on their assessment (EVAL-1E) and focus on specific content and proposed indicators (see Section  5.3.2). The word cloud also allows gaining an impression that does not consider thematic connections between more or less potentially misleading and non-misleading tweets. However, users (such as EVAL-1G, EVAL-1I or EVAL-2P) identified which topics were important in Twitter communication regarding the COVID-19 pandemic and, engaging with the list of tweets, also expanded their focus onto characteristics of misleading tweets. Based on the two-step evaluation, we interpret users’ choices as also depending on their personal preferences (Adikari et al., 2006; Huang et al., 2018). This includes considering the specific crisis context, in which anxiety is relatively prevalent (Ahmad & Murad, 2020). One individual engaged in heuristic processing (Hong & Kim, 2020) in the course of focusing on a specific misleading post:

“*This [tweet; AN], for example, by this guy is [...]. It does not make sense; it’s a pandemic and no conspiracy*” (EVAL-2M).

Offering diverse options, including an overview of sensitive contents proves beneficial due to its level of abstraction.

#### Equality

To make the web app more accessible, we chose conventional colors (Heeks & Ospina, 2018; Huang et al., 2018), which users highlighted positively. For example, the “*Twitter-like blue*” (EVAL-1E) or differentiated coloring of misleading (red) and valid (green) information (EVAL-1C) was noted. One participant noted being slightly color blind but that they did not require an alternative color scheme. Another participant with relative high media literacy proposed integrating a dark color mode (EVAL-1A). This was appreciated by one expert user in the second evaluation:

“*I’m a simple man. I see Dark Theme, I press activate. That’s how it is; of course [it’s] a ‘must have’ for any web app*”(EVAL-2K).

**Fig. 5:**
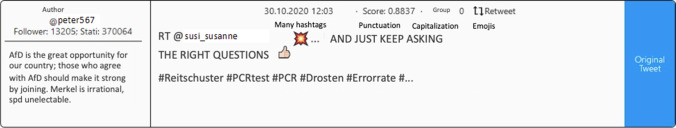
Evaluation 1: The indicators are at the top; the button has a text description

**Fig. 6:**
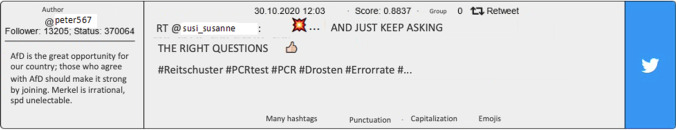
Evaluation 2: The indicators are at the bottom; the button has a Twitter icon

**Fig. 7:**
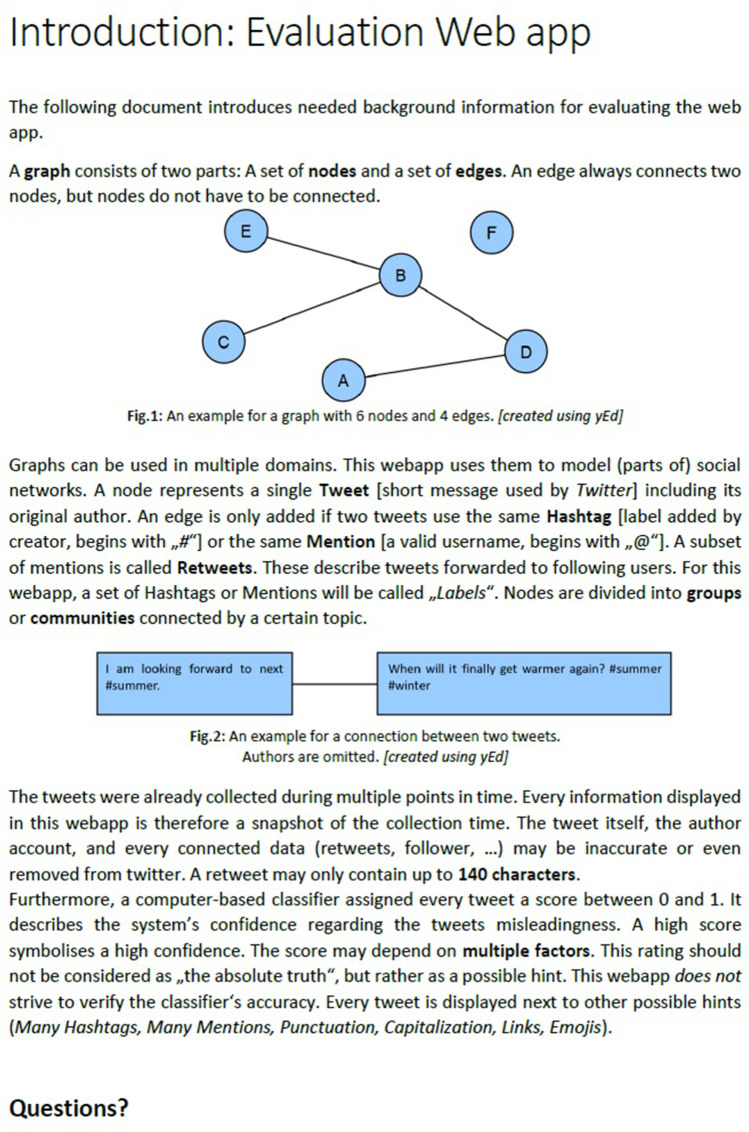
Introductory flyer which was shared with participants after a short exploration phase

**Fig. 8:**
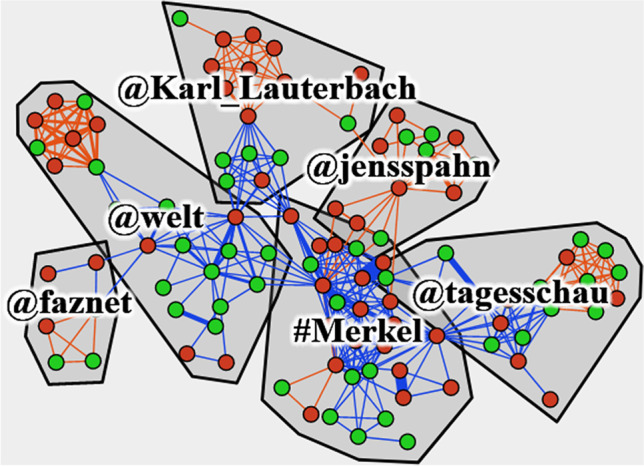
A network with thematic labels, referring to commonly used hashtags and mentions. They represent politicians as well as traditional media

**Fig. 9:**
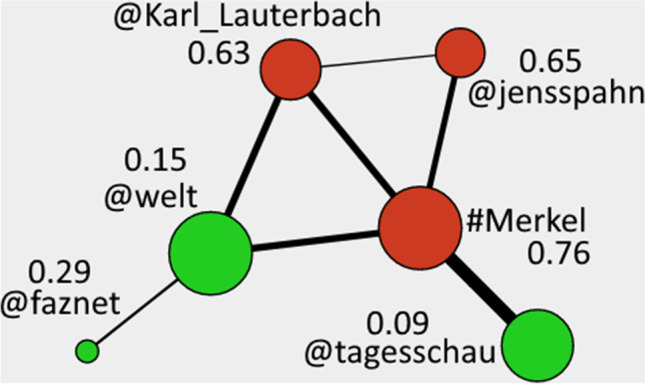
Alternative visualization with most popular themes based on the selected data set and indication of scores

Even though it “*took a short moment to find their way around*”, operation was “*mostly intuitive*” (EVAL-1G). To reflect a more entertaining environment (Micallef et al., 2021; Huang et al., 2018), we also included a “play” button into the generation UI. We further tried to refrain from symbols which may not be well-known and instead relied on text. Similarly, we also reduced the transparent display of technical terms such as “upload CSV” as it caused information overload in the first evaluation (EVAL-1B, EVAL-1C, EVAL-1D, EVAL-1E) (Hosseini et al., 2017). However, the friendly and partially well-known (Huang et al., 2018) twitter icon was preferred over description of “original tweet” (see Figs. 5, 6) which is indicated by positive feedback in the second evaluation (EVAL-2L).

One may also consider that non-expert users are not aware of the meaning of hashtags (#) or mentions (@) (EVAL-2P); yet, as most users who may rely on the web app may have a registered Twitter account and to reduce potential overload, we did not change the interface in this regard. As Twitter language or slang was identified as a barrier to lay users, we displayed the indicators more separately at the bottom of the Section (see Figs. 5, 6). Across both evaluations, expert users approached the “threshold” and showed interest in further details. In contrast, lay users rather refrained from exploring the slider freely but kept it in the middle or chose extremes on the continuum (EVAL-1C). In sum, all participating individuals were able to conduct the task of creating a network and thus could increase their competencies. They also showed interest to engage with the topic prospectively.

## Discussion

In the following, we communicate relevant implications with respect to enhancing learning, with the latter being understood to contribute to resilient socio-technical information systems in contexts where a lot of information is disseminated with high frequency and, paired with heterogeneous information quality, may overwhelm individuals (see Section 6.1). As social media has been increasingly used more broadly, people with different motivations, levels of media literacy, and attitudes use platforms for information retrieval. Insights apply specifically to human-social media information systems but also more generally when humans use search engines for different purposes (Koltay, 2012). Subsequently, we point out limitations and potential future work (see Section 6.2).

### Implications for IS Research on Learning and Resilience in Contexts of Unreliable Information Exchange

Based on our proposed artifact which relies on SNA and the BERT classifier, we can formulate implications which contribute to the knowledge base dedicated to enhancing learning processes when a vast amount of potentially unreliable information is disseminated. Considering design requirements (see Table 2), we reflect on our findings with regard to learning, rapidity, diversity & flexibility, transparency, and equality.

*(1) Focus on important system features which structure communication permits a deeper understanding of how information is produced.*

As identified by Goldberg (2021) and Lewandowsky and van der Linden (2021), countermeasures which aim at “societal inoculation” are more sustainable compared to interventions which relate to singular posts and require shorter attention spans (Pennycook et al., 2021; Pennycook & Rand, 2019). Further, as the latter may be perceived by users as categorizing posts simplistically without any explanatory information (Kirchner & Reuter, 2020), people may not have the possibility to train their analytical skills as much as they could. As indicated by the evaluation, participants with different backgrounds in media literacy and use were eager to learn about different narratives on social media and patterns which can give orientation to information processing on the Internet. Using a platform’s relevant system features as a frame of reference for presenting social media data makes sense as they organize online communication. Emphasis on their structuring function by building the summarizing network on them allows users to reflect on how content is produced and how social media works. To users who have prior knowledge about social media, presentation of social media content by network visualization appears “intuitively” (Naumann et al., 2007). This allows for further learning processes as cognitive capacities can be used for acquiring more detailed, new knowledge.

*(2) “Slowing down” information processing allows to pay attention to posts in the first place but should not decrease user motivation.*

Cost-sensitive approaches have been proposed as advantageous (Thirumuruganathan et al., 2021). In the context of the high speed of information dissemination on social media, users expect an efficient information retrieval for various purposes. Additionally, platform providers have an interest in satisfying user needs, which makes it difficult to create conditions of foundational learning. However, we designed a web app which affords exploration (in contrast to, e.g., warning symbols) and followed Heeks and Ospina (2018) in prioritizing learning as a requirement. This was done because prior literature emphasized the problem of short attention spans and the potential of foundational learning (Pennycook et al., 2021; Micallef et al., 2021). Additionally, we aimed for rapidity which was also tested for in the evaluation. Non-representative results suggested that, after improving the design, participants were faster. Nevertheless, the iterative evaluation did not indicate that participants of the first round were more impatient and showed decreased motivation as it took longer on average. Understanding motivation to be a dynamic variable (Huang et al., 2018), it is important to guarantee a satisfactory user experience, even when topics are more serious.

*(3) A diverse and flexible system can lead to information overload considering heterogeneous user groups and the subjective dimension of transparency.*

Existing studies on interpretability of indicators (Hartwig & Reuter, 2019; Wanas et al., 2008) and transparency in information systems (Hosseini et al., 2017; Kahn et al., 2002) already point out the importance of transparency, implying information quality, to learning. As noted by Hosseini et al. (2017), transparency can also lead to information overload, which was validated in the web app’s evaluation. Participants with higher media literacy wished for more explanation on the BERT classifier whereas non-expert users were at times overwhelmed by the different interface components. We tried to include these to offer a diverse and flexible design to users considering the objective to resonate with all the heterogeneous backgrounds, user habits and preferences and thus *added* features instead of focusing on how to satisfy intersecting needs and goals. Dynamic networks which allow for zoom-ins on different nodes and clusters already offer a great flexibility which enables users equally to learn and explore information on the different levels of abstraction. However, there is always a trade-off between diversity and transparency when a variety of people use the information system and information quality needs to be assured.

### Limitations & Outlook

Similar to Hoaxy (Shao et al., 2018), the proposed web app operates, with reference to source tweets, in parallel with Twitter. Prospectively, the thematic network may be integrated into the Twitter UI via a plug-in such as TrustyTweet (Hartwig & Reuter, 2019). In this case, future research may set up a different experimental environment and rely on near real-time data from the Twitter API instead of pre-established datasets whose selection required some costly effort by participants. Additional parameters such as custom search terms could be integrated then as well. Further, the two-step evaluation was conducted with a relatively small number of participants and thus offers non-representative findings. As one participant indicated to have slight dyschromatopsia but did not have any barriers in identifying features, complementing works may also consider generalizable results regarding issues of equality, i.e., accessibility and inclusion (Heeks & Ospina, 2018). For systematic testing, user modelling which considers different motivations and subsequent purposeful sampling of participating users is essential and may complement our illustrative work. Our work contributes to existing research on diverse approaches to counter the spread of misinformation and, following parameters of digital resilience in IS research (Heeks & Ospina, 2018) as well as including transparency (Hosseini et al., 2017), emphasizes important considerations for design of user-centered tools that encourage social media users’ media literacy. In principle, the web app is context-independent and may rely on near real-time data input. Instead of prior works which have proposed user networks (Milani et al., 2020; Ahmed et al., 2020; Memon & Carley, 2020), we focus on topics indicated by hashtags, mentions, and retweets. In our view, this implies a plausible shift from personalized accounts to thematic narratives which are to be targeted when dealing with misleading information. However, visualizations may be suggestive, proposing connections to be based on actual interactions between authors of tweets. Displaying relevant labels aims to counteract such impressions. Future works may further investigate relationships among requirements and also consider the impact of individual information processing on collectively used systems.

## Conclusion

The proposed intervention poses a first approach to counter the spread of misinformation with a special focus on increasing digital resilience. Thereby, our design science approach, comprising conceptualization and implementation of a web app as well as a two-step evaluation by *Think Aloud* studies, focuses on the design validation of a resilient information system. In this regard, we consider the relationship between individual human users and the social media platform Twitter and evaluate its potential for digital resilience based on tweets regarding the COVID-19 pandemic in Germany. Considering events such as the “storming of the Reichstag building” (Tagesschau, 2020) in August, 2020, we identify an “infodemic” (Ghebreyesus, 2020) as a problem which can be approached by offering an assisting tool. Although some participants were overwhelmed at first, the flexible and diverse web app allows for learning. The latter is fundamental to establish digital resilience and aims at shifting user focus onto thematic trends and relevant system functions which help in assessing disseminated information more analytically. Additionally, proposed “white-box” indicators enable reflection about classification of information. Relying on the BERT classifier (Devlin et al., 2019), we do not suggest that information can always be wrong or right depending on the perspective. Rather, the respective threshold aims to emphasize that some information holds more misleading (or more accurate) elements than other and that is is relevant for individual users to assess content critically. Adding to the knowledge base, our approach contributes with a reflection on attributes of resilience as well as on potentials and limitations of information systems aiming to tackle the spread of misinformation.

## Appendix

Tables 6, 7 and Figs. 7, 8, 9.

**Table 6:** Participants from both evaluations. Personal information is based on their own statements. Subjects who use Twitter as a news medium or consume conventional media but are active on Twitter are categorized as having a high level of media literacy. Motivation reflects participants’ interest in the issue of misinformation and the COVID-19 pandemic

Participants	Media Use	Motivation	Twitter Use	Media
				Literacy
1A, b. *1999*	social media, conventional (Twitter, online newspaper)	entertaining	registered account	high
1B, b. *1992*	conventional (newspaper apps)	casual consumption of news	no contact	low
1C, b. *1958*	conventional (newspaper)	professional (physician)	rare contact	low
1D, b. *2001*	conventional (newspaper apps)	no interest in fake news	rare contact	low
1E, b. *1994*	social media (Twitter)	consumption limited to 15 minutes a day	active for 2.5 years	high
1F, b. *1996*	conventional (newspaper)	studies	registered account	high
1G, b. *1997*	social media, conventional (newspaper apps, Twitter)	studies	active for 1 year	high
1H, b. *1997*	social media, conventional (Twitter, online newspaper)	current reporting	active for 8 years	
1I, b. *2001*	social media (online portals)	works at testing site, “frustrated” by breaking news	contact w/ embedded tweets in online articles	high
2J, b. *1992*	conventional (newspaper apps)	familiarization at the beginning	“second hand”	low
2K, b. *2000*	conventional, social media (online newspaper, Twitter)	passive, recommendations from friends	active for 6 years	high
				high
2L, b. *1963*	conventional (online newspaper)	passive consumption	no contact	low
2M, b. *2001*	conventional (online news sites)	annoyed by breaking news	consuming without account	high
2N, b. *1996*	conventional (news apps, newspaper)	studies, news	active for 10 years	high
2O, b. *1996*	conventional (news apps)	studies	active for 1.5 years	high
2P, b. *1962*	conventional (news sites)	entertainment (fake news), general interest	no contact	low

**Table 7:** Outline of the evaluation. Starting with checking individuals’ media literacy and personal motivation, participants conducted a phase of autonomous exploration in which they articulated their thoughts and assessed retrospectively. As a third step, they were introduced to social network analysis by the researcher and conducted a standardized task. Concluding the session, semi-structured interviews allowed participants to reflect on specific experiences and give feedback openly. We also made *changes* between the first and the second phase of evaluation

**1.**	**Media Literacy, Background**
a.	What is your year of birth?
b.	What is your main source for news?
c.	Are you interested in fake news or COVID-19?
	How do you engage with these topics?
d.	Did you create a Twitter account?
	Are you actively using it?
	If so, since about when?
	If not, do you still meet tweets in your daily life?
**2.**	**Exploration, Independent Orientation**
a.	Please navigate the webapp on your own and get used to the general layout.
	You are welcome to experiment with any feature.
	Please try to think aloud.
b.	What is your opinion on the general design?
c.	Which part made a negative impression on you?
	What would you change?
d.	Which part made a positive impression on you?
e.	Were you able to orient yourself? Is the data presentation intuitive?
f.	*If used, what is your opinion on the dark color mode?*
**3.**	**Task, Use Cases**
	[An introduction (see Fig. 7) about networks and the approach is presented.]
	The following tasks require you to think aloud as well.
a.	Please create a new network with a maximum number of 150 nodes in the period from 10/30/2020-10/31/2020. The network should contain retweets but not single nodes. Connections should not be created using the topic “CORONA/COVID”.
b.	What is the main topic (besides COVID)?
c.	Please browse through all tweets containing “#Merkel” to find possible indicators for misleading content. You may use every displayed information.
d.	*Please search about 3 tweets.*
**4.**	**Conclusive Interview**
a.	Did the introduction help you to understand the web app?
	Is this kind of insight important to you?
b.	Is the shown information comprehensible and supportive?
c.	You may browse the web app again. Are you missing some information (e.g., about the classifier) or would you remove some parts?
d.	Did you learn new possible indicators for misinformation?
e.	Which additional function could be useful?
	*What is your opinion on the Topic-Button?*
f.	Do you have any final remarks?

## References

[CR1] Adikari, S., McDonald, C., & Collings, P. (2006). A design science approach to an hci research project. In *Proceedings of the 18th Australia conference on computer-human interaction: Design: Activities, artefacts and environments*, OZCHI ’06 (pp. 429–432). Association for Computing Machinery. 10.1145/1228175.1228265

[CR2] Ahmad, A. R., & Murad, H. R. (2020). The impact of social media on panic during the covid-19 pandemic in Iraqi Kurdistan: Online questionnaire study. *Journal of Medical Internet Research, 22*(5). 10.2196/19556 10.2196/19556 PMC7238863 32369026

[CR3] Ahmed, W., Vidal-Alaball, J., Downing, J., & López Seguí, F. (2020). Covid-19 and the 5g conspiracy theory: Social network analysis of twitter data. *Journal of Medical Internet Research, 22*(5). 10.2196/19458 10.2196/19458 PMC7205032 32352383

[CR4] Aswani, R., Kar, A. K., & Ilavarasan, P. V. (2019). Experience: Managing misinformation in social media—insights for policymakers from twitter analytics. *Journal of Data and Information Quality, 12*(1), 10.1145/3341107

[CR5] Bostock, M. (2020). D3.js. https://d3js.org/

[CR6] Castillo, C., Mendoza, M., & Poblete, B. (2011). Information credibility on twitter. In *Proceedings of the 20th international conference on World Wide Web*, (pp. 675–684)

[CR7] Clauset, A., Newman, M. E. J., & Moore, C. (2004). Finding community structure in very large networks. *Physical Review E, 70*(6). 10.1103/physreve.70.066111 10.1103/PhysRevE.70.066111 15697438

[CR8] Clayton, K., Blair, S., Busam, J., Forstner, S., Glance, J., Green, G., et al. (2020). Real solutions for fake news? measuring the effectiveness of general warnings and fact-check tags in reducing belief in false stories on social media. *Political Behavior,**42*. 10.1007/s11109-019-09533-0.

[CR9] CNBC (2021). Youtube to add labels to some health videos amid misinformation backlash. https://www.cnbc.com/2021/07/19/youtube-labeling-some-health-videos-amid-misinformation-backlash.html. Accessed 13 Jan 2022

[CR10] CNN (2021). Reddit takes action against groups spreading covid misinformation. https://edition.cnn.com/2021/09/01/tech/reddit-covid-misinfor mation-ban/index.html. Accessed 13 Jan 2022

[CR11] Comes T, Meesters K, Torjesen S (2017). Making sense of crises: the implications of information asymmetries for resilience and social justice in disaster-ridden communities. Sustainable and Resilient Infrastructure.

[CR12] Courchesne, L., Ilhardt, J., & Shapiro, J. (2021). Review of social science research on the impact of countermeasures against influence operations. Harvard Kennedy School Misinformation Review 10.37016/mr-2020-79

[CR13] Cui, L., Shu, K., Wang, S., Lee, D., & Liu, H. (2019). Defend: A system for explainable fake news detection. In *Proceedings of the 28th ACM international conference on information and knowledge management*, CIKM ’19 (pp. 2961–2964). New York: Association for Computing Machinery 10.1145/3357384.3357862

[CR14] Dailey, D., & Starbird, K. (2015). “it’s raining dispersants”: Collective sensemaking of complex information in crisis contexts. In *Proceedings of the 18th ACM conference companion on computer supported cooperative work & social computing*, CSCW’15 Companion (pp. 155–158). New York: Association for Computing Machinery 10.1145/2685553.2698995

[CR15] Dailey D, Starbird K (2014). Journalists as crowdsourcerers: Responding to crisis by reporting with a crowd. Computer Supported Cooperative Work (CSCW).

[CR16] de Cock Buning, M. (2018). A multi-dimensional approach to disinformation. Report of the independent High level Group on fake news and online disinformation. Tech. rep. 10.2759/739290

[CR17] Desvars-Larrive A, Dervic E, Haug N, Niederkrotenthaler T, Chen J, Di Natale A, Lasser J, Gliga DS, Roux A, Sorger J (2020). A structured open dataset of government interventions in response to covid-19. Scientific Data.

[CR18] Devlin, J., Chang, M. W., Lee, K., & Toutanova, K. (2019). BERT: Pre-training of deep bidirectional transformers for language understanding. In *Proceedings of the 2019 Conference of the North American Chapter of the Association for Computational Linguistics: Human Language Technologies*, Volume 1 (Long and Short Papers), (pp. 4171–4186). Minneapolis, Minnesota: Association for Computational Linguistics 10.18653/v1/N19-1423

[CR19] Fonteyn M, Kuipers B, Grobe S (1993). A description of think aloud method and protocol analysis. Qualitative Health Research - QUAL HEALTH RES.

[CR20] Fromm, J., Eyilmez, K., Baßfeld, M., Majchrzak, T. A., & Stieglitz, S. (2021). Social media data in an augmented reality system for situation awareness support in emergency control rooms. *Information Systems Frontiers*. 10.1007/s10796-020-10101-9.

[CR21] García-Saiz D, Palazuelos C, Zorrilla M (2014). Data mining and social network analysis in the educational field: An application for non-expert users.

[CR22] Garista, P., & Pocetta, G. (2014). Digital resilience: meanings, epistemologies and methodologies for lifelong learning. 10.13140/2.1.3552.1605

[CR23] Gausen, A., Luk, W., & Guo, C. (2021). Can we stop fake news? using agent-based modelling to evaluate countermeasures for misinformation on social media

[CR24] Ghebreyesus, T. A. (2020). Munich security conference. https://www.who.int/dg/speeches/detail/munich-security-confer ence

[CR25] Goggins, S., Mascaro, C., & Mascaro, S. (2012). Relief work after the 2010 haiti earthquake: Leadership in an online resource coordination network. In *Proceedings of the ACM 2012 conference on computer supported cooperative work*, CSCW ’12 (pp. 57–66). New York: Association for Computing Machinery 10.1145/2145204.2145218

[CR26] Goldberg, B. (2021). Can “inoculation” build broad-based resistance to misinformation? https://medium.com/jigsaw/can-inoculation-build-broad-based-resistance-to-misinformation-6c67e517e314. Accessed 13 Jan 2022

[CR27] Google (2020). Flutter. https://flutter.dev/

[CR28] Goudalo, W., & Kolski, C. (2016). Towards advanced enterprise information systems engineering - solving resilience, security and usability issues within the paradigms of socio-technical systems. In *ICEIS*

[CR29] Gregor S, Hevner AR (2013) Positioning and presenting design science research for maximum impact. MIS Quarterly 37(2):337–355, http://www.jstor.org/stable/43825912

[CR30] Guardian (2020). Arizona man dies after attempting to take trump coronavirus ‘cure’. https://www.theguardian.com/world/2020/mar/24/coronavirus-cure-kills-man-after-trump-touts-chloroquine-phosphate. Accessed 25 Sep 2020

[CR31] Guess A, Lerner M, Lyons B, Montgomery J, Nyhan B, Reifler J, Sircar N (2020). A digital media literacy intervention increases discernment between mainstream and false news in the united states and india. Proceedings of the National Academy of Sciences.

[CR32] Hansson, S. O. (2017). Science denial as a form of pseudoscience. *Studies in History and Philosophy of Science Part A,**63,*. 10.1016/j.shpsa.2017.05.002. 10.1016/j.shpsa.2017.05.002 28629651

[CR33] Hartwig, K., & Reuter, C. (2019). TrustyTweet: An indicator-based browser-plugin to assist users in dealing with fake news on Twitter. In *Proceedings of the international conference on wirtschaftsinformatik (WI)* (pp. 1858–1869)

[CR34] Heeks, R., & Ospina, A. V. (2018). Conceptualising the link between information systems and resilience: A developing country field study. *Information Systems Journal,**29*. 10.1111/isj.12177.

[CR35] Hegner, M. (2003). *Methoden zur Evaluation von Software, IZ-Arbeitsbericht* (Vol. 29). Bonn: Informationszentrum Sozialwissenschaften.

[CR36] Hevner AR, March ST, Park J, Ram S (2004). Design science in information systems research. Management Information Systems Quarterly.

[CR37] Hong, H., & Kim, H. J. (2020). Antecedents and consequences of information overload in the covid-19 pandemic. *International Journal of Environmental Research and Public Health, 17*(24), 10.3390/ijerph17249305 10.3390/ijerph17249305 PMC7763334 33322715

[CR38] Hosseini M, Shahri A, Phalp K, Ali R (2017). Four reference models for transparency requirements in information systems. Requirements Engineering.

[CR39] Huang, Y. L., Starbird, K., Orand, M., Stanek, S. A., & Pedersen, H. T. (2015). Connected through crisis: Emotional proximity and the spread of misinformation online. In *Proceedings of the 18th ACM conference on computer supported cooperative work & social computing*, CSCW ’15 (pp. 969–980). New York: Association for Computing Machinery 10.1145/2675133.2675202

[CR40] Huang, S. Y., Yang, M. M., & Chen, C. H. (2018). When do motivational factors lead to negative user experience on social networking applications? *Australasian Journal of Information Systems,**22*. 10.3127/ajis.v22i0.1533.

[CR41] Instagram (2019). Combatting misinformation on instagram. https://about.instagram.com/blog/announcements/combatting-mis information-on-instagram. Accessed 13 Jan 2022

[CR42] Jo, W., Lee, J., Park, J., & Kim, Y. (2020). Online information exchange and anxiety spread in the early stage of the novel coronavirus (covid-19) outbreak in south korea: Structural topic model and network analysis. *Journal of Medical Internet Research, 22*(6), 10.2196/19455 10.2196/19455 PMC7268668 32463367

[CR43] Kahn B, Strong D, Wang R (2002). Information quality benchmarks: Product and service performance. Commun ACM.

[CR44] Kahne J, Bowyer B (2017). Educating for democracy in a Partisan Age: Confronting the challenges of motivated reasoning and misinformation. American Educational Research Journal.

[CR45] Kaufhold MA, Rupp N, Reuter C, Habdank M (2020). Mitigating information overload in social media during conflicts and crises: design and evaluation of a cross-platform alerting system. Behaviour & Information Technology.

[CR46] Kirchner, J., & Reuter, C. (2020). Countering fake news: A comparison of possible solutions regarding user acceptance and effectiveness. *CSCW 2020 - Proceedings of the 2020 ACM international conference on computer-supported cooperative work and social computing*

[CR47] Koltay T (2012). Information architecture, information overload, and the literacies. Journal of Information Architecture.

[CR48] Krause, N. M., Freiling, I., Beets, B., & Brossard, D. (2020). Fact-checking as risk communication: the multi-layered risk of misinformation in times of covid-19. *Journal of Risk Research*, 1–8, 10.1080/13669877.2020.1756385

[CR49] Leavitt, A., & Clark, J. A. (2014). Upvoting hurricane sandy: Event-based news production processes on a social news site. In *Proceedings of the SIGCHI conference on human factors in computing systems*, CHI ’14 (pp. 1495–1504). Association for Computing Machinery 10.1145/2556288.2557140

[CR50] Leavitt, A., & Robinson, J. J. (2017). The role of information visibility in network gatekeeping: Information aggregation on reddit during crisis events. In *Proceedings of the 2017 ACM conference on computer supported cooperative work and social computing*, CSCW ’17 (pp. 1246–1261). New York: Association for Computing Machinery 10.1145/2998181.2998299

[CR51] Lee YW, Strong DM, Kahn BK, Wang RY (2002). Aimq: a methodology for information quality assessment. Information & Management.

[CR52] Lehman, A., & Miller, S.J. (2020). A theoretical conversation about responses to information overload. *Information, 11*(8) 10.3390/info11080379

[CR53] Lewandowsky S, van der Linden S (2021). Countering misinformation and fake news through inoculation and prebunking. European Review of Social Psychology.

[CR54] McDougall J (2019). Media literacy versus fake news: critical thinking, resilience and civic engagement. Media Studies.

[CR55] Memon, S. A., & Carley, K. M. (2020). Characterizing covid-19 misinformation communities using a novel twitter dataset. arXiv:2008.00791

[CR56] Meta (2022). About fact-checking on facebook. https://en-gb.facebook.com/business/help/2593586717571940. Accessed 13 Jan 2022

[CR57] Micallef, N., Avram, M., Menczer, F., & Patil, S. (2021). Fakey: A game intervention to improve news literacy on social media. *Proceedings of the ACM on Human-Computer Interaction, 5*(CSCW1), 10.1145/3449080

[CR58] Mihailidis P, Viotty S (2017). Spreadable spectacle in digital culture: Civic expression, fake news, and the role of media literacies in “post-fact” society. American Behavioral Scientist.

[CR59] Milani E, Weitkamp E, Webb P (2020). The visual vaccine debate on Twitter: A social network analysis. Media and Communication.

[CR60] Misra S, Roberts P, Rhodes M (2020). Information overload, stress, and emergency managerial thinking. International Journal of Disaster Risk Reduction.

[CR61] Morris, M. R., Counts, S., Roseway, A., Hoff, A., & Schwarz, J. (2012). Tweeting is believing? understanding microblog credibility perceptions. In *Proceedings of the ACM 2012 conference on computer supported cooperative work*, CSCW ’12 (pp. 441–450). Association for Computing Machinery. 10.1145/2145204.2145274

[CR62] Müller G, Koslowski TG, Accorsi R, Abramowicz W (2013). Resilience - a new research field in business information systems?. Business information systems workshops.

[CR63] Naumann A, Hurtienne J, Israel JH, Mohs C, Kindsmüller MC, Meyer HA, Hußlein S, Harris D (2007). Intuitive use of user interfaces: Defining a vague concept. Engineering psychology and cognitive ergonomics.

[CR64] Nerghes, A., Kerkhof, P., & Hellsten, I. (2018). Early public responses to the zika-virus on youtube: Prevalence of and differences between conspiracy theory and informational videos. In *Proceedings of the 10th ACM Conference on Web Science*, WebSci ’18, (pp. 127–134). New York: Association for Computing Machinery 10.1145/3201064.3201086

[CR65] Nied, C., Stewart, L., Spiro, E., & Starbird, K. (2017). Alternative narratives of crisis events: Communities and social botnets engaged on social media. *CSCW 2017 - Companion of the 2017 ACM Conference on Computer Supported Cooperative Work and Social Computing*, (pp 263–266). 10.1145/3022198.3026307

[CR66] Paschalides, D., Kornilakis, A., Christodoulou, C., Andreou, R., Pallis, G., Dikaiakos, M. D., & Markatos, E. (2019). Check-it: A plugin for detecting and reducing the spread of fake news and misinformation on the web. arXiv:1905.04260

[CR67] Pennycook, G., & Rand, D. G. (2019). Lazy, not biased: Susceptibility to partisan fake news is better explained by lack of reasoning than by motivated reasoning. *Cognition,**188,* 39–50. 10.1016/j.cognition.2018.06.011, the Cognitive Science of Political Thought. 10.1016/j.cognition.2018.06.011 29935897

[CR68] Pennycook G, Epstein Z, Mosleh M, Arechar A, Eckles D (2021). Shifting attention to accuracy can reduce misinformation online. Nature.

[CR69] Pfeffers, K., Tuunanen, T., Gengler, C.E., Rossi, M., Hui, W., Virtanen, V., & Bragge, J. (2006). The design science research process: A model for producing and presenting information systems research. In *Proceedings of the first international conference on design science research in information systems and technology (DESRIST 2006)*, (pp. 83–106). Claremont, CA, USA,

[CR70] Python-Software-Foundation (2020). Python 3.9.1. https://www.python.org/downloads/release/python-391/

[CR71] Reuter, C., Kaufhold, M. A., Spielhofer, T., & Hahne, A. S. (2017). Social media in emergencies: A representative study on citizens’ perception in Germany. *Proceedings of the ACM on Human-Computer Interaction, 1*(CSCW). 10.1145/3134725

[CR72] Reuter, C., Ludwig, T., & Pipek, V. (2014). Ad hoc participation in situation assessment: Supporting mobile collaboration in emergencies. *ACM Transactions on Computer-Human Interaction, 21*(5), 10.1145/2651365

[CR73] Richards AS, Banas JA (2015). Inoculating against reactance to persuasive health messages. Health Communication.

[CR74] Roberts E, Farrington J, Skerratt S (2015). Evaluating new digital technologies through a framework of resilience. Scottish Geographical Journal.

[CR75] Roozenbeek J, van der Linden S (2018). The fake news game: actively inoculating against the risk of misinformation. Journal of Risk Research.

[CR76] Seaborn K, Chignell M, Gwizdka J (2021). Psychological resilience during covid-19: A meta-review protocol. BMJ Open.

[CR77] Sell T, Hosangadi D, Trotochaud M (2020). Misinformation and the us ebola communication crisis: Analyzing the veracity and content of social media messages related to a fear-inducing infectious disease outbreak. BMC Public Health.

[CR78] Shao, C., Hui, P. M., Wang, L., Jiang, X., Flammini, A., Menczer, F., & Ciampaglia, G. (2018). Anatomy of an online misinformation network. *PLoS ONE,**13,*. 10.1371/journal.pone.0196087. 10.1371/journal.pone.0196087 PMC5922526 29702657

[CR79] Sharma, K., Seo, S., Meng, C., Rambhatla, S., Dua, A., & Liu, Y. (2020). Coronavirus on social media: Analyzing misinformation in twitter conversations. arXiv:2003.12309

[CR80] Shrestha, A., Cater-Steel, A., & Toleman, M. (2014). How to communicate evaluation work in design science research? an exemplar case study. *25th Australasian conference on information systems*

[CR81] Shu, K., Bernard, H. R., & Liu, H. (2019). Studying fake news via network analysis: Detection and mitigation. In *Emerging research challenges and opportunities in computational social network analysis and mining* (pp. 43–65), 10.1007/978-3-319-94105-9_3, arXiv:1804.10233

[CR82] Shu K, Sliva A, Wang S, Tang J, Liu H (2017). Fake news detection on social media: A data mining perspective. SIGKDD Explor Newsl.

[CR83] SMT (2021). Tiktok adds new video warning labels to stop the spread of misinformation. https://www.socialmediatoday.com/news/tiktok-adds-new-video-warning-labels-to-stop-the-spread-of-misinformation/594481/. Accessed 13 Jan 2022

[CR84] Soden R, Palen L (2018). Informating crisis: Expanding critical perspectives in crisis informatics. Proceedings of the ACM on Human-Computer Interaction.

[CR85] Starbird, K. (2017). Examining the alternative media ecosystem through the production of alternative narratives of mass shooting events on twitter. In *Proceedings of the 11th international conference on Web and social media*, ICWSM 2017, (pp. 230–239). Montréal, Québec, Canada

[CR86] Starbird, K., & Palen, L. (2011). “voluntweeters”: Self-organizing by digital volunteers in times of crisis. *Proceedings of the international conference on human factors in computing systems*

[CR87] Starbird, K., Arif, A., & Wilson, T. (2019). Disinformation as collaborative work: Surfacing the participatory nature of strategic information operations. *Proceedings of the ACM on Human-Computer Interaction, 3*(CSCW). 10.1145/3359229

[CR88] Starbird, K., Maddock, J., Orand, M., Achterman, P., & Mason, R. M. (2014). Rumors, False Flags, and Digital Vigilantes: Misinformation on Twitter after the 2013 Boston Marathon Bombing. *IConference*. 10.9776/14308.

[CR89] Tagesschau (2020). Entsetzen über Eskalation am Reichstagsgebäude. https://www.tagesschau.de/inland/corona-demo-berlin-131.html. Accessed 16 Oct 2020

[CR90] Thirumuruganathan S, Simpson M, Lakshmanan LV (2021). To Intervene or Not To Intervene: Cost based intervention for combating fake news.

[CR91] Thuan NH, Drechsler A, Antunes P (2019). Construction of design science research questions. Communications of the Association for Information Systems.

[CR92] Tran, T., Valecha, R., Rad, P., & Rao, H. R. (2020). An investigation of misinformation harms related to social media during two humanitarian crises. *Information Systems Frontiers*, pp 1–9, 10.1007/s10796-020-10088-3 10.1007/s10796-020-10088-3 PMC7641657 33169067

[CR93] Twitter (2020). Updating our approach to misleading information. https://blog.twitter.com/en_us/topics/product/2020/updating- our-approach-to-misleading-information. Accessed 13 Jan 2022

[CR94] Twitter (2021). Introducing birdwatch, a community-based approach to misinformation. https://blog.twitter.com/en_us/topics/product/2021/introducing-birdwatch-a-community-based-approach-to-misinformation. Accessed 13 Jan 2022

[CR95] Vermeeren, A. P. O. S., Law, E. L. C., Roto, V., Obrist, M., Hoonhout, J., Väänänen-Vainio-Mattila, K. (2010). User experience evaluation methods: Current state and development needs. In *Proceedings of the 6th nordic conference on human-computer interaction: Extending Boundaries*, NordiCHI ’10 (pp. 521–530). ACM 10.1145/1868914.1868973

[CR96] Wanas, N., El-Saban, M., Ashour, H., & Ammar, W. (2008). Automatic scoring of online discussion posts. In *Proceedings of the 2nd ACM Workshop on Information Credibility on the Web*, WICOW ’08 (pp. 19–26). Association for Computing Machinery 10.1145/1458527.1458534

[CR97] Wong, B. (2011). Points of view: Color blindness. *Nature, 8*(441) 10.1038/nmeth.1618

[CR98] World Health Organization. (2020). Novel coronavirus (2019-nCoV) Situation Report, 13. Clarification, Clinical and Presentation, Clinical and Factors, Risk: Tech. Rep. February.

[CR99] Wu, L., & Liu, H. (2018). Tracing fake-news footprints: Characterizing social media messages by how they propagate. In *WSDM 2018 - Proceedings of the 11th ACM international conference on web search and data mining*, (pp. 637–645). Association for Computing Machinery, Inc. 10.1145/3159652.3159677

[CR100] Wulf, V., Rohde, M., Pipek, V., & Stevens, G. (2011). Engaging with practices: Design case studies as a research framework in cscw. pp 505–512. 10.1145/1958824.1958902

[CR101] Yadav, K. (2021). Platform interventions: How social media counters influence operations. https://carnegieendowment.org/2021/01/25/platform-interventions-how-social-media-counters-influence-operations-pub-83698. Accessed 14 Jan 2022

[CR102] Yan, R., Lapata, M., & Li, X. (2012). Tweet recommendation with graph co-ranking. In *Proceedings of the 50th annual meeting of the association for computational linguistics*, (pp. 516–525)

[CR103] Yang, J., & Lee, S. (2020). Framing the mers information crisis: An analysis on online news media’s rumour coverage. *Journal of Contingencies and Crisis Management*, pp 1–13, 10.1111/1468-5973.12292

[CR104] Zhou, X., & Zafarani, R. (2020). A survey of fake news: Fundamental theories, detection methods, and opportunities. *ACM Computing Surveys, 53*(5) 10.1145/3395046

